# microRNA-34a (miRNA-34a) Mediated Down-Regulation of the Post-synaptic Cytoskeletal Element SHANK3 in Sporadic Alzheimer's Disease (AD)

**DOI:** 10.3389/fneur.2019.00028

**Published:** 2019-02-06

**Authors:** Yuhai Zhao, Vivian R. Jaber, Ayrian LeBeauf, Nathan M. Sharfman, Walter J. Lukiw

**Affiliations:** ^1^LSU Neuroscience Center, Louisiana State University Health Sciences Center New Orleans, New Orleans, LA, United States; ^2^Department of Anatomy and Cell Biology, Louisiana State University Health Sciences Center, New Orleans, LA, United States; ^3^Department of Ophthalmology, Louisiana State University Health Sciences Center New Orleans, New Orleans, LA, United States; ^4^Department of Neurology, Louisiana State University Health Sciences Center New Orleans, New Orleans, LA, United States

**Keywords:** Alzheimer's disease (AD), miRNA-34a, neurotransmission, post-synaptic density proteins, SHANK3 protein, superior temporal lobe neocortex (Brodmann A22), synaptic structure in disease, synaptic transmission

## Abstract

Integrating a combination of bioinformatics, microRNA microfluidic arrays, ELISA analysis, LED Northern, and transfection-luciferase reporter assay data using human neuronal-glial (HNG) cells in primary culture we have discovered a set of up-regulated microRNAs (miRNAs) linked to a small family of down-regulated messenger RNAs (mRNAs) within the superior temporal lobe neocortex (Brodmann A22) of sporadic Alzheimer's disease (AD) brain. At the level of mRNA abundance, the expression of a significant number of human brain genes found to be down-regulated in sporadic AD neocortex appears to be due to the increased abundance of a several brain-abundant inducible miRNAs. These up-regulated miRNAs—including, prominently, miRNA-34a—have complimentary RNA sequences in the 3′ untranslated-region (3′-UTR) of their target-mRNAs that results in the pathological down-regulation in the expression of important brain genes. An up-regulated microRNA-34a, already implicated in age-related inflammatory-neurodegeneration–appears to down-regulate key mRNA targets involved in synaptogenesis and synaptic-structure, distinguishing neuronal deficits associated with AD neuropathology. One significantly down-regulated post-synaptic element in AD is the proline-rich SH3 and multiple-ankyrin-repeat domain SHANK3 protein. Bioinformatics, microRNA array analysis and SHANK3-mRNA-3′UTR luciferase-reporter assay confirmed the importance of miRNA-34a in the regulation of SHANK3 expression in HNG cells. This paper reports on recent studies of a miRNA-34a-up-regulation coupled to SHANK3 mRNA down-regulation in sporadic AD superior-temporal lobe compared to age-matched controls. These findings further support our hypothesis of an altered miRNA-mRNA coupled signaling network in AD, much of which is supported, and here reviewed, by recently reported experimental-findings in the scientific literature.

## Overview

Alzheimer's disease (AD) is a complex, insidious, and ultimately lethal neurodegenerative disorder characterized by the appearance of pro-inflammatory lesions known as senile plaques and neurofibrillary tangles and a progressive disruption of the synaptic architecture of the brain. Synaptic loss and synapto-axonal pathology in AD is thought to be the strongest correlation to, and the basis for, the loss of sensory, intellectual, and cognitive function in AD patients (1–5). Indeed the most recent studies employing stepwise regression analysis has revealed that the major correlate of cognitive deficiency in AD is synaptic loss in the prefrontal cortex, and this contributes strongly to the association between global psychometric assessment and neuronal network collapse (4–7).

Because inter-neuronal signaling in the human central nervous system (CNS) is achieved through a complex network of presynaptic and postsynaptic elements essential in the conveyance of both electrical and neurochemical information, we have focused our investigations on the structural and functional integrity of key pre- and post-synaptic components in the superior temporal lobe neocortex (Brodmann A22), an anatomical region targeted by the AD process. One recently characterized core element essential for the efficient operation of this complex inter-neuronal signaling network is the relatively abundant ~185 kDa proline-rich cytoskeletal scaffolding and post-synaptic density (PSD) protein known as SHANK3 (SH3-and multiple ankyrin repeat domains 3; encoded at human chr 22q13.33) (8, 8–13). Interestingly, disruption in the abundance of the postsynaptic SHANK3 cytoskeletal anchoring protein has been associated with neurological disorders including autism spectrum disorder (ASD), bipolar disorder (BD), Phelan-McDermid syndrome (PMS; 22q13.3 deletion syndrome), intellectual disability, schizophrenia (SZ), and sporadic AD (9, 10, 14). In this “*Perspectives*” article, we review and comment on recent advances in SHANK3 research as it pertains to age-related neurodegeneration using AD as an important example wherever possible. We also include some original data that provides evidence indicating that SHANK3 is under post-transcriptional control by an inducible NF-kB-regulated microRNA-34a in the temporal lobe neocortex, and adds to the growing list of pathological genetic mechanisms and cardiovascular and neurological disease-relevant messenger RNAs (mRNAs) targeted by the CNS-abundant miRNA-34a [see below;(15–18)].

## SHANK3 Down-Regulation and Synaptic Signaling Deficits

**Figure 1:**
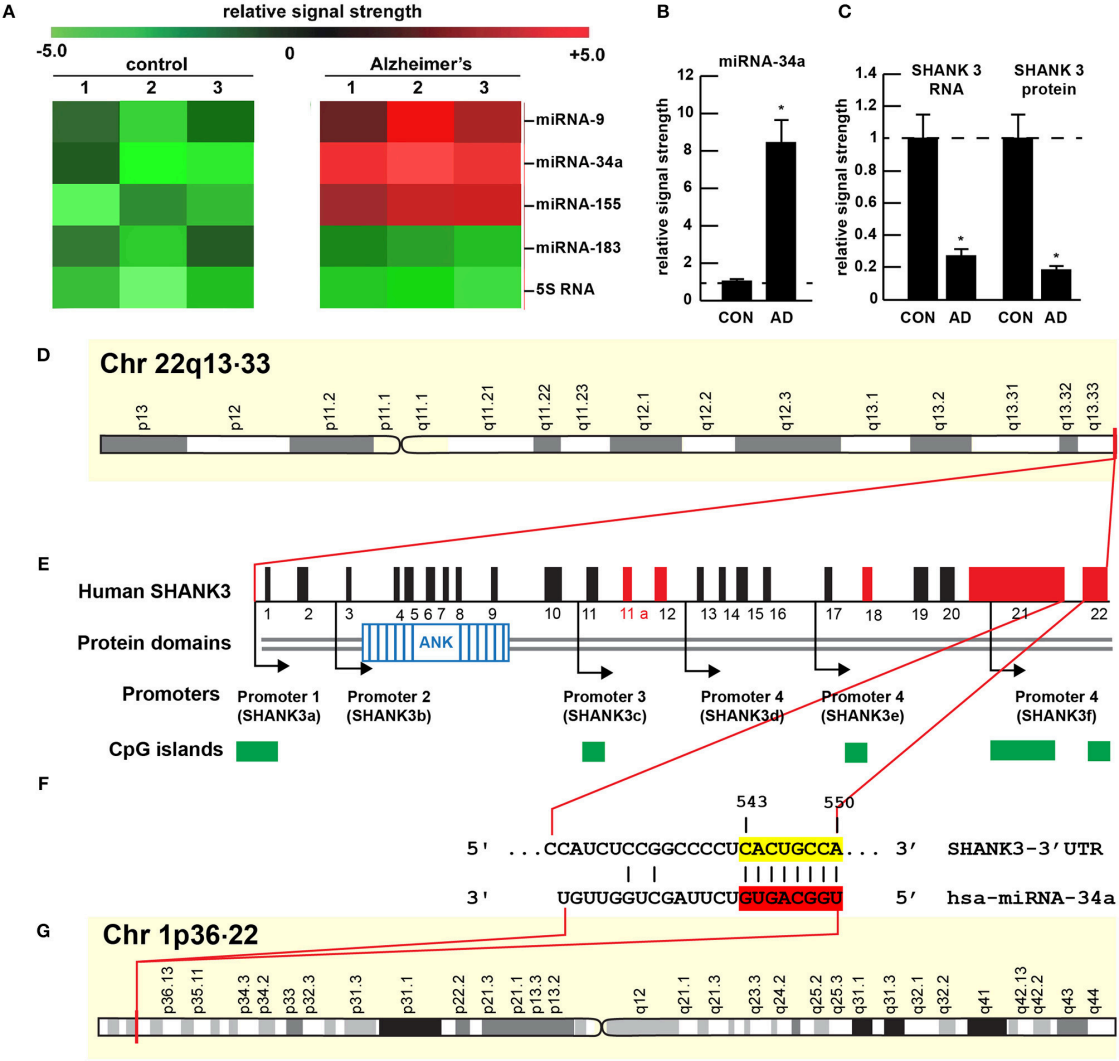
Gene products on human chromosomes 1 and 22 interactively contribute to SHANK3 expression in CNS tissues. **(A)** Results of miRNA microfluidic array analysis; miRNA-34a is significantly up-regulated in the sporadic AD temporal lobe compared to miRNA-183 and 5S RNA control sncRNA markers; the numbers 1–3 indicates 3 separate control and age-matched AD cases; all female; control mean age 72.1 ± 6.6 years; AD mean age 73.8 ± 8.2 years; *N* = 3 control and 3 AD: all post mortem intervals <3 h; **(B)** quantitation of miRNA-34a levels in bar graph format; **(C)** quantitation of SHANK3 mRNA (using Northern analysis) and SHANK3 protein (using ELISA) in the superior temporal lobe of control and AD as previously described (9, 23); *N* = 3; *p < 0.001 (ANOVA). **(D–G)** highly schematicized depiction of the human SHANK3 gene organization at chr 22q13.33 and the human miRNA-34a gene organization at chr 1p36.22; with a P_**CT**_ (probability of conserved targeting) or Friedman score of 83 [(24); that has been calculated for all highly conserved miRNA families]; this hsa-miRNA-34a-SHANK3-3′-UTR recognition/interaction is highly favorable and almost certain to occur in the cytoplasm/nucleoplasm of CNS cells; **(E)** has been modified from (25); the homo sapien (hsa) microRNA-34a (hsa-miRNA-34a) is encoded from the distal end of human chromosome 1p (at chr 1p36.22) and generates a 22 nt mature miRNA-34a species; hence the expression of at least two genes, one cytoskeletal and structural (SHANK3) and one regulatory (miRNA-34a) on human chromosomes 1p and 22q is required for regulating the expression of SHANK3.

The SH3 and multiple ankyrin (ANK)-repeat domain-proteins SHANK1, SHANK2, and SHANK3 (also known as the ProSAP family, SHANK postsynaptic density proteins, the proline-rich synapse-associated family of proteins; also known as PROSAP2, PSAP2, SCZD15, SPANK-2) encode a small family of related postsynaptic scaffolding proteins that are highly abundant at glutamatergic synapses in the human CNS (8, 12). SHANK proteins are essential to post-synaptic structure and function in connecting, linking, networking and anchoring neurotransmitter receptors, ion channels, and other integral membrane proteins to the actin cytoskeleton and in the normal “homeostatic” operation of G-protein-coupled signaling pathways. Research evidence indicates that the massive SHANK3 protein (at ~185 kDa) forms an extensive post-synaptic cytoskeletal scaffolding network (involving the linkage of multiple SHANK3 proteins at the PSD) to which the smaller PSD-95 (at ~95kDa) protein is tethered usually via the SAPAP protein (~100 kDa); interestingly both SHANK3 and PSD-95 proteins, highly interactive components of the PSD complex, are reduced in abundance in the temporal lobe of AD-affected brain (13, 19, 20). SHANK3 post-synaptic scaffolding proteins thereby play essential roles in synapse formation and organization, synaptic cell adhesion, dendritic spine maturation and synaptic vesicle release (4, 11, 13, 21, 22). All SHANK species are abundantly expressed in the human CNS but exhibit different anatomical, developmental, and spatial patterns of expression; SHANK3 appears to have preferential expression in the human neocortex and hippocampus. Indeed, like all SHANK proteins, SHANK3 contains multiple domains for extensive protein-protein interaction including ankyrin (ANK) repeats—hence a deficit in these central and major cytoskeletal components, key players for both synapse formation and the modulation of synaptic transmission and synaptic plasticity, may be responsible for major synaptic aberrations and loss of the capability for inter-neuronal communication, with ensuing cognitive impairment, as has been observed in multiple neurological disorders (9, 10, 12) (Figures 1A–E). As for mentioned, these disorders include several seemingly unrelated human neurological syndromes such as sporadic AD, ASD, BD, PMS, and SZ (8, 9, 21). Indeed, synaptic dysfunction and abnormal behaviors in transgenic murine models are apparent in mice lacking adequate SHANK3; in the CNS of the transgenic AD (TgAD) 5x familial AD murine model engineered to overexpress the 42 amino acid amyloid-beta (Aβ42) peptide, SHANK3 was also found to be significantly downregulated but the pathological mechanisms remain unclear (9, 10, 12, 21, 25–27). Interestingly, the extra-neural levels of Aβ peptide oligomers have been shown to strongly correlate with the severity of cognitive impairment (using the Blessed information-memory-concentration score and mini-mental state examination, MMSE) and with the loss of synaptic markers such as SHANK3 that results in the disruption of synaptic function (5, 28). It has been known for some time that the application of known pro-inflammatory stressors, such as the Aβ42 peptide and neurotoxic metal sulfates (such as aluminum sulfate) to human neuronal-glial (HNG) cells in primary culture also results in a significant decrease in the expression of SHANK3 (9, 10, 12). Collectively these data indicate that deficits in SHANK3-expression may be one common denominator linking a wide-range of human neurodegenerative disorders that exhibit a progressive synaptic disorganization temporally associated with progressive, developmental, and/or age-related intellectual disability combined with sensory and cognitive decline.

## miRNA-34a

**Figure 2:**
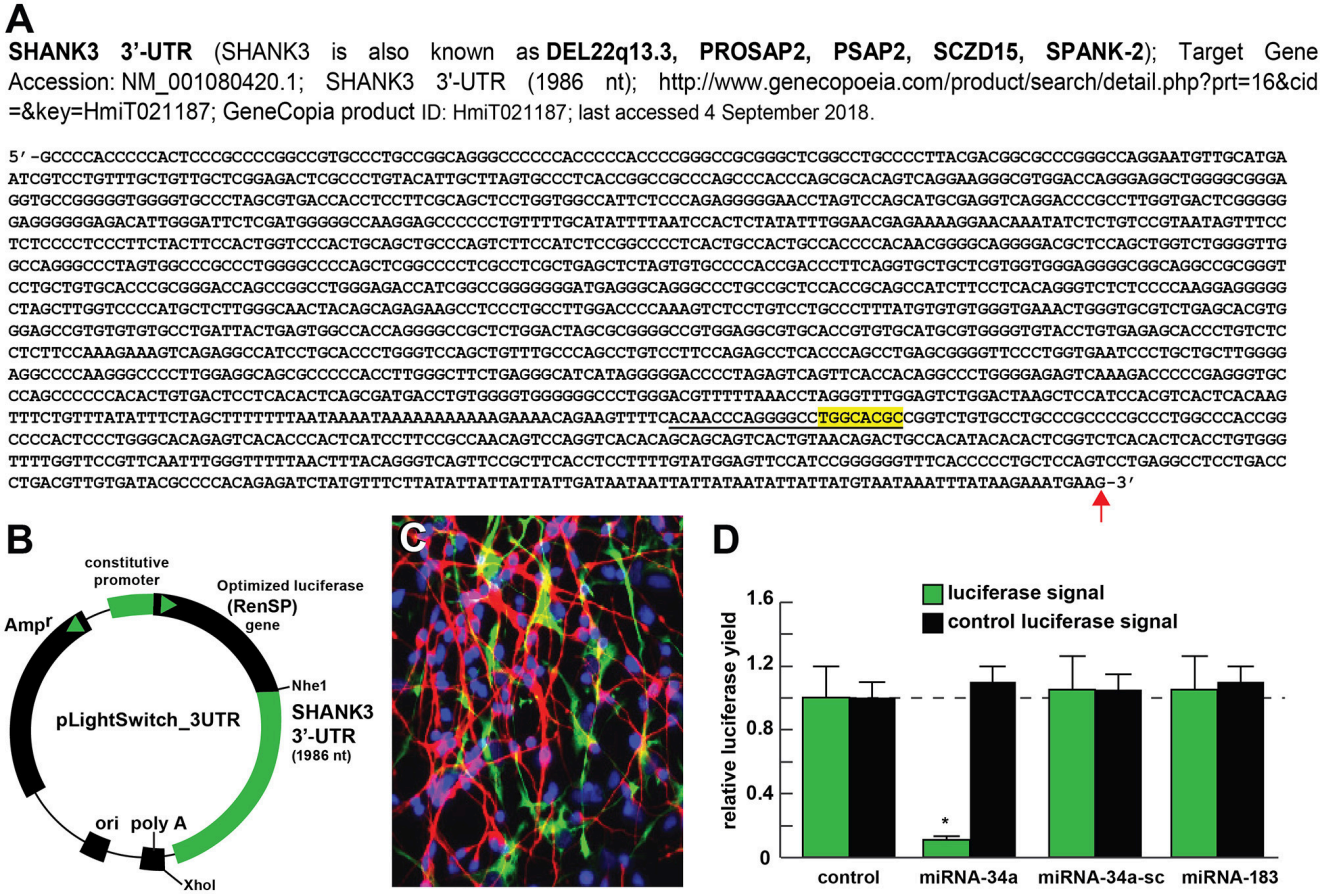
Luciferase reporter vector-based studies of miRNA-34a and SHANK3 expression - Functional validation of a miRNA-34a-SHANK3–3′UTR interaction. **(A)** partial ribonucleotide sequence of the 1986 nt SHANK3-mRNA-3′-UTR is shown in the 5′-3′ direction; the 22 nucleotide (nt) miRNA-34a-SHANK3-3′UTR complementarity-interaction region is indicated by a black underline and the 8 nt SHANK3-mRNA-3′-UTR seed sequence is overlaid in yellow; a single vertical red arrow indicates the 5′ end of a poly A+ tail in the SHANK3 mRNA; the SHANK3 mRNA sequence derived from NM_018965; **(B)** SHANK3-mRNA-3′UTR expression vector luciferase reporter assay (pLightSwitch-3′UTR; Cat#S801178; Switchgear Genomics, Palo Alto CA); in this vector, the entire 1986 nucleotide SHANK3 3′UTR was ligated into the unique Nhe1-Xho1site; not drawn to scale; **(C)** HNG cells, 2 weeks in primary culture; neurons (red stain; λ_max_ = 690 nm), DAPI (blue nuclear stain; λ_max_ = 470 nm) and glial fibrillary associated protein (GFAP; glial-specific green stain; λ_max_ = 520 nm); the HNG cell culture is about 60% confluent and at 2 weeks of culture contains 70% neurons and 30% astroglia; human neurons do not culture well in the absence of glia; neurons also show both extensive arborization and display electrical activity (unpublished; Lonza); 40X magnification; HNG cells were transfected with the SHANK3-mRNA-3′UTR expression vector luciferase reporter were treated exogenously with a stabilized miRNA-34a, a scrambled control miRNA-34a (miRNA-34a-sc) or control miRNA-183; see references and text for further details; **(D)** compared to control, HNG cells transfected with a scrambled (sc) control pLightSwitch-3'UTR vector, the SHANK3-mRNA-3′UTR vector exhibited decreased luciferase signal to a mean of 0.16-fold of controls in the presence of miRNA-34a; this same vector exhibited no change in the presence of the control miRNA-34a-sc or miRNA-183; for each experiment (using different batches of HNG cells) a control luciferase signal was generated and included separate controls with each analysis; in addition a control vector β-actin-3′UTR showed no significant effects on the relative luciferase signal yield after treatment with either miRNA-183 or miRNA-34a (data not shown); a dashed horizontal line set to 1.0 is included for ease of comparison; *N* = 6; *p < 0.001 (ANOVA). The results suggest a physiologically relevant miRNA-34a-SHANK3-mRNA-3′UTR interaction and a miRNA-34a-mediated down-regulation of SHANK3 expression in HNG cells. This pathogenic interaction may be related to the down-regulation of other immune, inflammatory, and synaptic system genes by up-regulated miRNAs in the CNS resulting in an impairment in trans-synaptic signaling and synaptic cytoarchitecture.

The 22 nucleotide (nt) miRNA-34a (Figures 1F, 2) encoded in humans as a single copy gene at chr1p36.22 has about ~1,200 predicted human mRNA targets using standard bioinformatics analysis (miRBase; EMBL-EBI; www.genecards.org/cgi-bin/carddisp.pl?gene=MIR34A; accessed 17 January 2019); major bioinformatics- and experimentally-verified miRNA-34a-mRNA targets include those encoding TREM2, a transmembrane glycoprotein of microglial cells that plays a role in amyloid sensing and removal (29). miRNA-34a has also been implicated in epithelial cell proliferation, in endothelial cell-mediated inflammation, in T-cell activation and in the regulation of the innate-immune system, in the down-regulation of the apoptosis regulator/suppressor Bcl-2, in both cardiovascular and neurovascular disease mechanisms involving epithelial and endothelial cell linings, and in the down-regulation of expression of specific synaptic cytoskeletal elements including SHANK3 [this publication (8, 9, 15–18)]. Recent data have further indicated that as an NF-kB-inducible microRNA, miRNA-34a appears to play analogous roles in AD, age-related macular degeneration (AMD), autism and in transgenic murine models of AD or AMD (TgAD, TgAMD) (18, 29–31). For example, miRNA-34a up-regulation in a double transgenic mouse model (APPswe/PSDeltaE9) of AD has been shown to inhibit the translation of the anti-apoptosis regulating protein Bcl-2 resulting in a progressive and pro-inflammatory neurodegeneration, and excessive miRNA-34a has a significant inhibitory effect on retinal pigment epithelial cell proliferation and migration (17, 30–32). More recently, miRNA-34a has been shown to regulate the calcium- and calmodulin-dependent serine/threonine protein phosphatase heterodimer calcineurin 1 to modulate endothelial inflammation and T-cell activation in the innate-immune system (33). We have observed a significant increase in miRNA-34a abundance in AD temporal lobe neocortex averaging a remarkable 8.1-fold increase over control in 6 AD brain tissue samples over control coupled to SHANK3 deficits, at both the SHANK3 mRNA level (to 0.27-fold of controls) and at the SHANK3 protein level (to 0.18-fold of controls) within the same neocortical tissue sample (Figures 1A–C). Taken together the results suggest that increases in miRNA-34a linked to SHANK3 decreases orchestrate a complex pathological program involving pro-inflammatory degeneration, endothelial and epithelial cell deficits, pro-apoptotic signaling and synaptic insufficiency in the aging human cardiovascular, neurovascular, and neurological systems.

## miRNA-34a Interactions With the SHANK3 mRNA 3′-UTR

The ~7,413 nt human SHANK3 mRNA [major species SHANK3a; GenBank: AB569469.1; https://www.ncbi.nlm.nih.gov/nuccore/AB569469.1;https://www.genecards.org/cgi-bin/carddisp.pl?gene=SHANK3]^1^ encoded at the distal arm of human chromosome 22q (chr 22q13.33) and spliced together from a 22 exon gene includes multiple anykyrin (ANK) repeat domains and a 1986 nt 3′-UTR with multiple binding sites for miRNA-34a (from position 543–550 and 549–556 of the 22 nt miRNA-34a; Figures 1D–G). Interestingly these miRNA-34a binding sites have been previously shown to be immediately flanked downstream by a single miRNA-146a binding site (23); miRNA-146a is a pro-inflammatory microRNA also linked to AD neuropathology and pro-inflammatory neurodegeneration (34, 35). Hence the SHANK3 mRNA-3′UTR provides a classic example of multiple miRNAs—and in this case multiple pro-inflammatory miRNAs (miRNA-34a and miRNA-146a)– targeting the same mRNA 3′-UTR; these miRNA-mRNA recognition features for SHANK3 are shared by *Homo sapiens*, chimpanzee and Rhesus monkey (http://www.targetscan.org/cgi-bin/targetscan/vert71/viewgene.cgi? members = miR-34-5p/449-5p&show cnc = 0&shownc = 0&subset = 1; accessed 17 January 2019) (23). Different SHANK3 gene deletions, duplications, and point mutations are also associated with ASD, intellectual disability, SZ, BD, and attention deficit hyperactivity disorder (ADHD) and these different genetic alterations may contribute to the pathophysiological and phenotypic diversity of neurological disorders related to SHANK3 gene mutations (14, 22, 36). Interestingly, multiple promoters for the human SHANK3 gene, often immediately associated with CpG islands, encode multiple SHANK3 species including SHANK3a, SHANK3b; SHANK3c, SHANK3d, SHANK3e, and SHANK3f (see Figure 1E); the significance of these 5 SHANK3 mRNA subspecies, all smaller than the full length SHANK3a mRNA, is not well understood but they may be involved in neuronal and synaptic development in different anatomical regions of the CNS (10, 14, 26, 37)

## Unanswered Questions

While a considerable amount of scientific evidence suggests that miRNA-34a (and miRNA-146a) are involved in progressive and ultimately lethal degenerative pathologies in human neurological, cardiovascular and neurovascular disease, at this point in time we cannot exclude the pathological participation of other miRNA species, other small non-coding RNAs (sncRNAs) or other pathological factors in the regulation of SHANK3 expression (23). There are currently ~2,654 known human miRNAs (http://mirbase.org/help/FAQs.shtml) but only about 30-35 miRNAs appear to be abundant in the human brain neocortex, hippocampus and retina (15, 23, 38). It will be interesting to see if miRNA-34a and miRNA-146a compete to control SHANK3-3′UTR binding and hence, ultimately SHANK3 expression and the status of the synaptic signaling network in health and in the SHANK3-mediated neuronal network collapse as typified in AD. The significance (if any) of adjacent and overlapping miRNA binding sites in the same 3′UTR is not well understood; it may be a built-in redundancy in the intrinsic miRNA-mRNA signaling system encoded on at least 2 chromosomes to ensure, for example, miRNA-34a-SHANK3 mRNA regulatory control. Very recently it has been established that the gastrointestinal (GI) tract microbiome may provide a long list of pro-inflammatory genetic mediators that are capable of transiting the aging GI tract and blood-brain barriers to upregulate a select number of pro-inflammatory miRNAs that have strong potential to induce neurological disease via the targeting of genes involved in the cytoskeleton, in the synapse, in the transit of signaling molecules across endothelial cell barriers and in the innate-immune response (5, 28, 34, 35, 39–44).

## Conclusions

There is a remarkable amount of pathological damage in the sporadic AD brain, including the progressive and simultaneous appearance of senile plaques and neurofibrillary tangles, neuronal atrophy, the appearance of inflammatory markers and extensive synaptic disruption. Indeed a significant number of studies have indicated both a progressive and overwhelming deficit in synaptic cytoarchitecture and synaptogenesis occurs in the AD-affected brain. Our studies indicate that deficits in the SHANK3 cytoskeletal post-synaptic protein, with resulting disruption in synaptic structure and function may be mediated at least in part by inducible NF-kB regulated pro-inflammatory microRNAs such as miRNA-34a. Lastly, AD is an extremely heterogeneous disease with multiple and often strongly inter-linked pathological deficits. For example Aβ42 peptide abundance, inflammatory degeneration, loss of SHANK3 and synaptic disruption all occur concurrently, especially in the moderate-to-latter stages of AD. It would be interesting to see if Aβ42 peptide levels, miRNA-34a-mediated SHANK3 abundance and synaptic deficits could be managed early during the course of the disease for therapeutic benefit. These data continue to indicate that increases in specific miRNAs coupled to deficits in SHANK3-expression may be one common denominator linking a wide-range of human neurological disorders that exhibit a progressive or developmental synaptic disorganization that is temporally associated with both intellectual disability and progressive cognitive decline.

## Ethics Statement

All human brain tissue acquisition and handling procedures involving postmortem human tissues were carried out in strict accordance with the ethics review board policies at donor institutions. The work in this investigative study was approved by the Institutional Biosafety Committee/Institutional Review Board (IBC/IRB) at the LSU Health Sciences Center, New Orleans LA 70112 USA.

## Author Contributions

YZ, VJ, AL, NS, and WL: collected, analyzed, and summarized the current SHANK3 literature. YZ, VJ, AL, and NS: performed the experiments and data extraction. WL: wrote the article.

### Conflict of Interest Statement

The authors declare that the research was conducted in the absence of any commercial or financial relationships that could be construed as a potential conflict of interest.

## Footnotes

^1^SHANK3 gene expression data:https://www.genecards.org/cgi-bin/carddisp.,pl?gene=SHANK3 (2018)
